# Acylated and unacylated ghrelin inhibit atrophy in myotubes co-cultured with colon carcinoma cells

**DOI:** 10.18632/oncotarget.20531

**Published:** 2017-08-24

**Authors:** Xianliang Zeng, Sizeng Chen, Yang Yang, Zhao Ke

**Affiliations:** ^1^ Department of Gastrointestinal Surgery, The First Affiliated Hospital of Fujian Medical University, Fuzhou, Fujian 350005, China

**Keywords:** cancer cachexia, ghrelin, co-culture, calpain, myotube

## Abstract

Cancer cachexia is a result of increased protein degradation and decreased protein synthesis. The multifunctional circulating hormone ghrelin promotes synthesis and inhibits degradation of muscle protein, but its mechanism of action is not fully understood. Here, we investigated whether co-culturing C2C12 myotubes with CT26 colon carcinoma cells induces myotube atrophy, and whether acylated ghrelin (AG) and unacylated ghrelin (UnAG) had anti-atrophic effects. We found that co-culture induced myotube atrophy and increased tumor necrosis factor-alpha (TNF-α) and myostatin concentrations in the culture medium. Moreover, co-culture down-regulated myogenin and MyoD expression, inhibited the Akt signaling, up-regulated ubiquitin E3 ligase expression, and activated the calpain system and autophagy in myotubes. Both AG and UnAG inhibited these changes. Our study describes a novel *in vitro* model that can be employed to investigate cancer cachexia, and our findings suggest a possible use for AG and UnAG in treating cancer cachexia.

## INTRODUCTION

Cancer cachexia is a multifactorial metabolic syndrome characterized by muscle wasting (with or without fat wasting), systemic inflammation, and a progressive loss of muscle function [1]. Cachexia occurs in ∼ 85% of terminal cancer patients, reduces the patient's tolerance and response to cancer treatment, and is responsible for ∼ 20% of all cancer deaths [2]. The pathogenesis of cancer cachexia is not completely understood; furthermore, conventional nutritional support does not fully reverse cancer cachexia, and no effective therapies currently exist [3]. Cancer cachexia-induced muscle wasting is a result of increased muscle proteolysis and decreased protein synthesis. While the ubiquitin-proteasome system [4], autophagy-lysosome system [5], myostatin pathway [6], and PI3K/AKT pathway [7] all play critical roles in muscle wasting, their involvement in cancer cachexia is not fully understood.

Ghrelin is a multifunctional circulating peptide hormone composed of 28-amino acids that exists in acylated (AG) and unacylated (UnAG) forms. The only structural difference between these forms, which originate from the same precursor, is that Ser 3 of AG is octanoylated by intracellular ghrelin-O-acyltransferase during post-translational processing. Both AG and UnAG are synthesized predominantly in stomach cells and then secreted into blood serum [8, 9]. Accumulating evidence indicates that ghrelin receptors are widely expressed in the central nervous system, intestine, pancreas, liver, adipose tissue, skeletal muscle, and cardiac muscle. The ghrelin system is therefore thought to play important roles in numerous biological functions, including appetite regulation, gastric motility, pancreatic function, metabolism, cardiovascular function, immune function, and muscle mass regulation, in both humans and animals [9–11]. Furthermore, both AG and UnAG might inhibit muscle wasting caused by aging, thermal injury, heart failure, chronic renal failure, cancer, and chemotherapy-induced cachexia by increasing muscle protein synthesis and decreasing proteolysis [12]. *In vitro* and *in vivo* studies have shown that both AG and UnAG can reduce pro-inflammatory cytokine levels [13], suppress the transcription factors forkhead box O3A (FoxO3a) and NF-κB, inhibit ubiquitin E3 ligases [9, 14], and regulate the PI3K/AKT/mTOR pathway and autophagy [15, 16]. However, the mechanisms underlying these effects remain unclear and require further investigation.

Calpains are a group of 14 calcium-activated cysteine proteases, including two ubiquitously expressed members, μ-calpain and m-calpain, and one muscle-specific member, calpain-3 [17]. While calpains are inactive under basal conditions, they can be activated by calcium and phospholipids. Calpastatin is the only known ubiquitously-expressed endogenous calpain inhibitor and is thus another important regulator of the calpain system. Calpains are implicated in several diseases, including muscular dystrophy, neurological disorders, and diabetes [18]. Furthermore, active calpain can anchor myofilaments to the Z disc, disrupt the structural integrity of myofilaments, and release actin and myosin for ubiquitination and degradation in animals with muscle atrophy [19]. Because the ubiquitin-proteasome system cannot degrade intact myofilaments, calpain-dependent cleavage of myofilaments is considered the initial step in myofilament degradation and plays a critical role in muscle wasting. Furthermore, active calpain can promote muscle atrophy by inhibiting Akt activity [20] and activating FoxO3a, NF-κB, and ubiquitin E3 ligases [19, 21, 22]. A recent study [23] in our laboratory confirmed that an activated calpain system, as indicated by an increased calpain/calpastatin ratio, contributed to skeletal muscle wasting in cachexic tumor-bearing mice, and calpain inhibitors reversed this effect.

Whether ghrelin affects the calpain system in skeletal muscle has not yet been investigated. In this study, we used a transwell-plate system to develop a novel myotube-carcinoma cell co-culture model. This model allows myotubes and carcinoma cells to grow in the same culture medium and permits intercellular communication without physical contact. We evaluated whether this model could be used to simulate the cancer cachexia environment and to induce myotube atrophy *in vitro*. We also investigated whether AG or UnAG inhibited myotube atrophy and the possible mechanisms involved, with a focus on the role of the calpain system.

## RESULTS

### Myotube diameter, MHC2/MHC7 levels, and MHC mRNA expression

**Figure 1:**
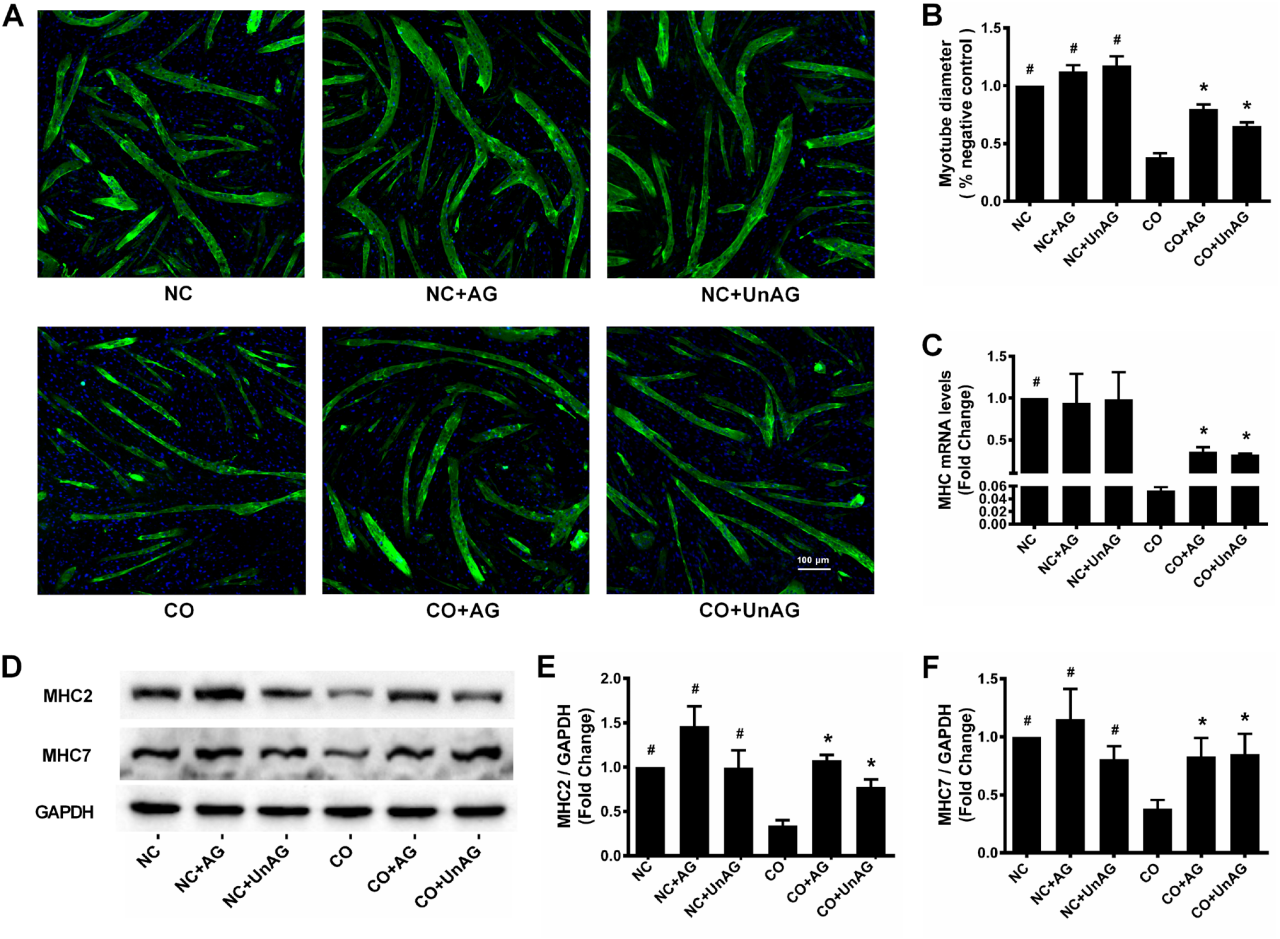
AG/UnAG prevents co-cultured myotubes breakdown **(A)** Immunofluorescence staining for anti-myosin heavy chain (MHC) antibody in C2C12 myotubes (× 100). MHC staining outlines the myotubes (green). 4′6-Diamidino-2-phenylindole was used to stain nuclei (blue). **(B)** Myotubes diameter expressed as % of negative control. Significant differences were detected between CO and any NC groups (^#^P < 0.001), between CO and CO+AG/UnAG groups (*P < 0.001), by one-way ANOVA followed by Tukey test. **(C)** The levels of MHC mRNA in C2C12 myotubes. mRNA levels were normalized to GAPDH and expressed as fold change from nagative control. Significant differences were detected between NC and CO groups (^#^P < 0.001), between CO and CO+AG/UnAG groups (*P = 0.044, P = 0.001; respectively), by one-way ANOVA followed by Dunnett's T3 test. **(D)** Western blot of MHC2, MHC7 and GAPDH in C2C12 myotubes. **(E)** Quantification of MHC2 was normalized to GAPDH. Significant differences were detected between CO and any NC groups (^#^P = 0.001, P < 0.001; P = 0.001 respectively), between CO and CO+AG/UnAG groups (*P < 0.001, P = 0.015; respectively), by one-way ANOVA followed by Tukey test. **(F)** Quantification of MHC7 was normalized to GAPDH. Significant differences were detected between CO and any NC groups (^#^P = 0.004, P = 0.001; P = 0.047 respectively), between CO and CO+AG/UnAG groups (*P = 0.035, P = 0.026; respectively), by one-way ANOVA followed by Tukey test. Data are represented as mean ± SD. Scale bar represents 100 μm.

After 24 hours of co-culture, myotube diameter decreased by nearly 60% in the CO group compared to the NC group (*P* < 0.001), and AG/UnAG prevented this decrease (*P* < 0.001); no significant differences were observed between the NC, NC+AG, and NC+UnAG groups (Figure 1A and 1B). Western blot results showed that co-culture decreased myotube MHC2 and MHC7 levels compared to those in the CO and NC groups. AG/UnAG also prevented this decrease, and no significant differences were observed between the NC, NC+AG, and NC+UnAG groups (Figure 1D-1F). Consistent with the western blot results, RT-qPCR demonstrated that co-culture downregulated MHC mRNA expression, and AG/UnAG prevented this downregulation (Figure 3C).

### Co-culture medium analysis

**Figure 2:**
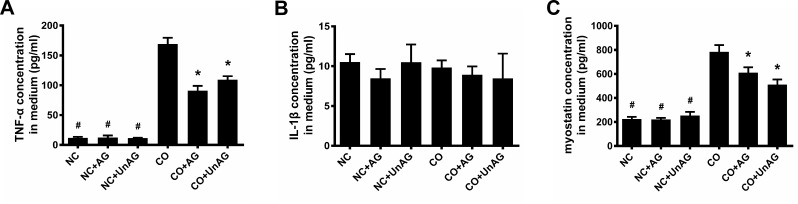
Concentrations of pro-cachexic factors in cell culture medium **(A)** ELISA for TNF-α concentrations in coculture medium. Significant differences were detected between CO and any NC groups (^#^P < 0.001), between CO and CO+AG/UnAG groups (*P < 0.001), by one-way ANOVA followed by Tukey test. **(B)** ELISA for IL-1β concentrations in coculture medium. No significant differences were detected among the six groups, by one-way ANOVA followed by Tukey test. **(C)** ELISA for myostatin concentrations in coculture medium. Significant differences were detected between CO and any NC groups (^#^P < 0.001), between CO and CO+AG/UnAG groups (*P = 0.001, P < 0.001; respectively), by one-way ANOVA followed by Tukey test. Data are represented as mean ± SD.

**Figure 3:**
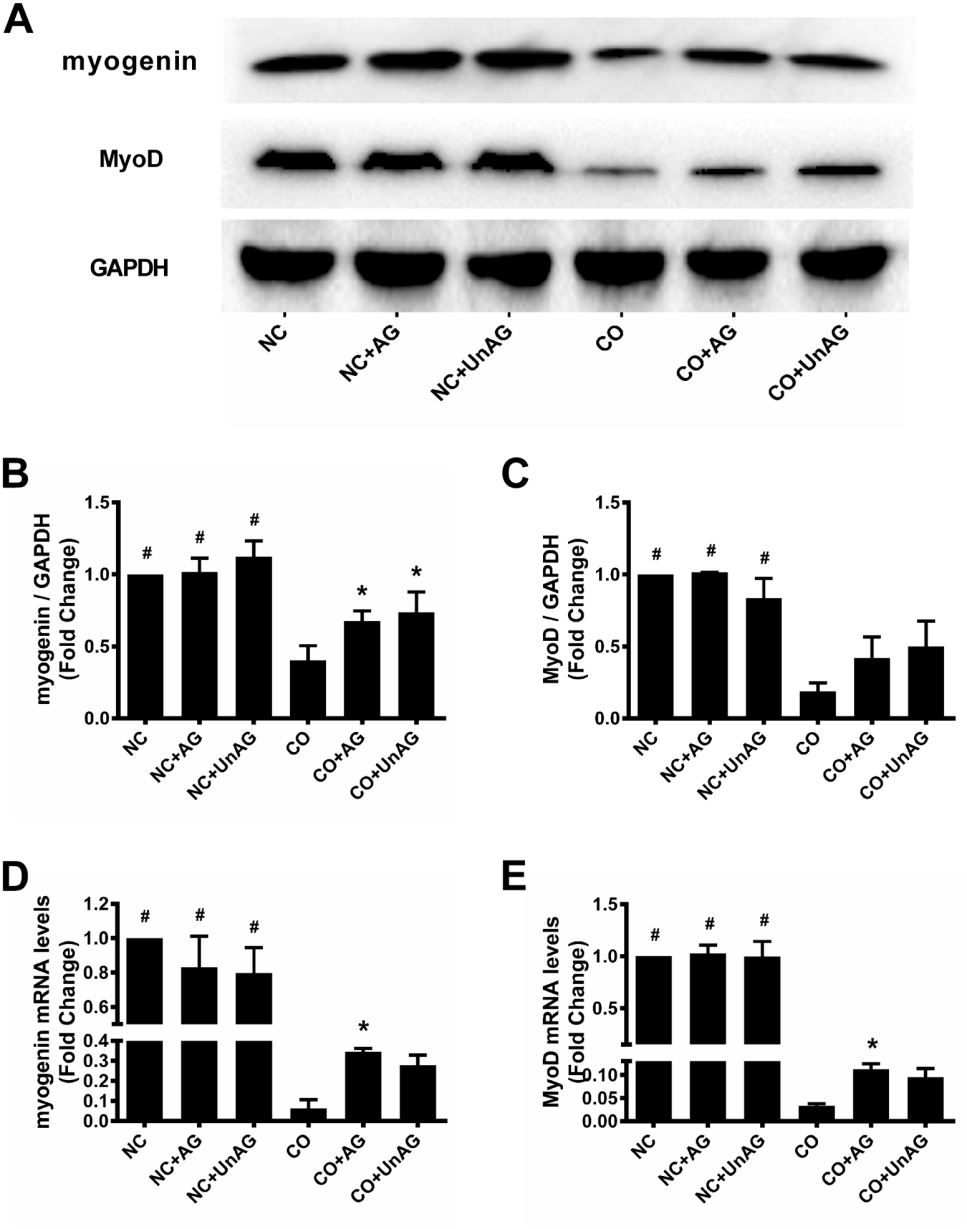
AG/UnAG improves pro-myogenesis transcription factors in co-cultured myotubes **(A)** Western blot of myogenin, MyoD and GAPDH in C2C12 myotubes. **(B)** Quantification of myogenin was normalized to GAPDH. Significant differences were detected between CO and any NC groups (^#^P < 0.001), between CO and CO+AG/UnAG groups (*P = 0.044, P = 0.012; respectively), by one-way ANOVA followed by Tukey test. **(C)** Quantification of MyoD was normalized to GAPDH. Significant differences were detected between CO and any NC groups (^#^P = 0.008, P = 0.008; P = 0.039 respectively), by one-way ANOVA followed by Dunnett's T3 test. **(D)** The levels of myogenin mRNA in C2C12 myotubes. mRNA levels were normalized to GAPDH. Significant differences were detected between NC and CO groups (^#^P < 0.001), between CO and CO+AG groups (*P = 0.039), by one-way ANOVA followed by Tukey test. **(E)** The levels of MyoD mRNA in C2C12 myotubes. mRNA levels were normalized to GAPDH. Significant differences were detected between NC and CO groups (^#^P < 0.001, P < 0.009; P = 0.031 respectively), between CO and CO+AG groups (*P = 0.016), by one-way ANOVA followed by Dunnett's T3 test. Data are represented as mean ± SD.

The above results indicate that co-culture of C2C12 myotubes with CT26 cells resulted in myotube atrophy, and AG/UnAG prevented this change. To assess whether myotube atrophy was induced by paracrine factors released from CT26 cells and whether AG/UnAG affected these paracrine factors, we measured concentrations of the atrophy-inducing cytokines TNF-α and IL-1β in the culture medium using ELISA assays [24]. As shown in Figure 2A, co-culture increased TNF-α concentration in the medium approximately 14-fold, and AG/UnAG prevented this increase. However, there were no differences in IL-1β concentrations among the six groups (Figure 3B).

Myostatin, a secreted growth factor belonging to the TGF-β-superfamily that inhibits myogenesis [25], was also detected in the medium. As shown in Figure 2C, co-culture significantly increased myostatin concentrations in the medium (*P* < 0.001), and AG/UnAG prevented this increase (*P* < 0.01). No significant differences in myostatin concentration were detected among the NC, NC+AG, and NC+UnAG groups.

### Myogenin and MyoD expression

We performed western blot and RT-qPCR studies to determine whether co-culture affected the expression of the muscle-specific myogenesis mediators myogenin and MyoD and the effects of AG/UnAG on their levels in myotubes. As shown in Figure 3A, 3B and 3D, co-culture decreased myogenin mRNA and protein levels in myotubes, and AG/UnAG prevented this decrease in all groups with the exception of myogenin mRNA levels in the CO+UnAG group (*P* = 0.154). Co-culture also decreased MyoD mRNA and protein levels in myotubes; while AG/UnAG tended to prevent this decrease in all groups, the effect was statistically significant only for MyoD mRNA level in the CO+AG group (*P* = 0.016) (Figure 3A, 3C, and 3F).

### Myostatin/Smad3 pathway activity

**Figure 4:**
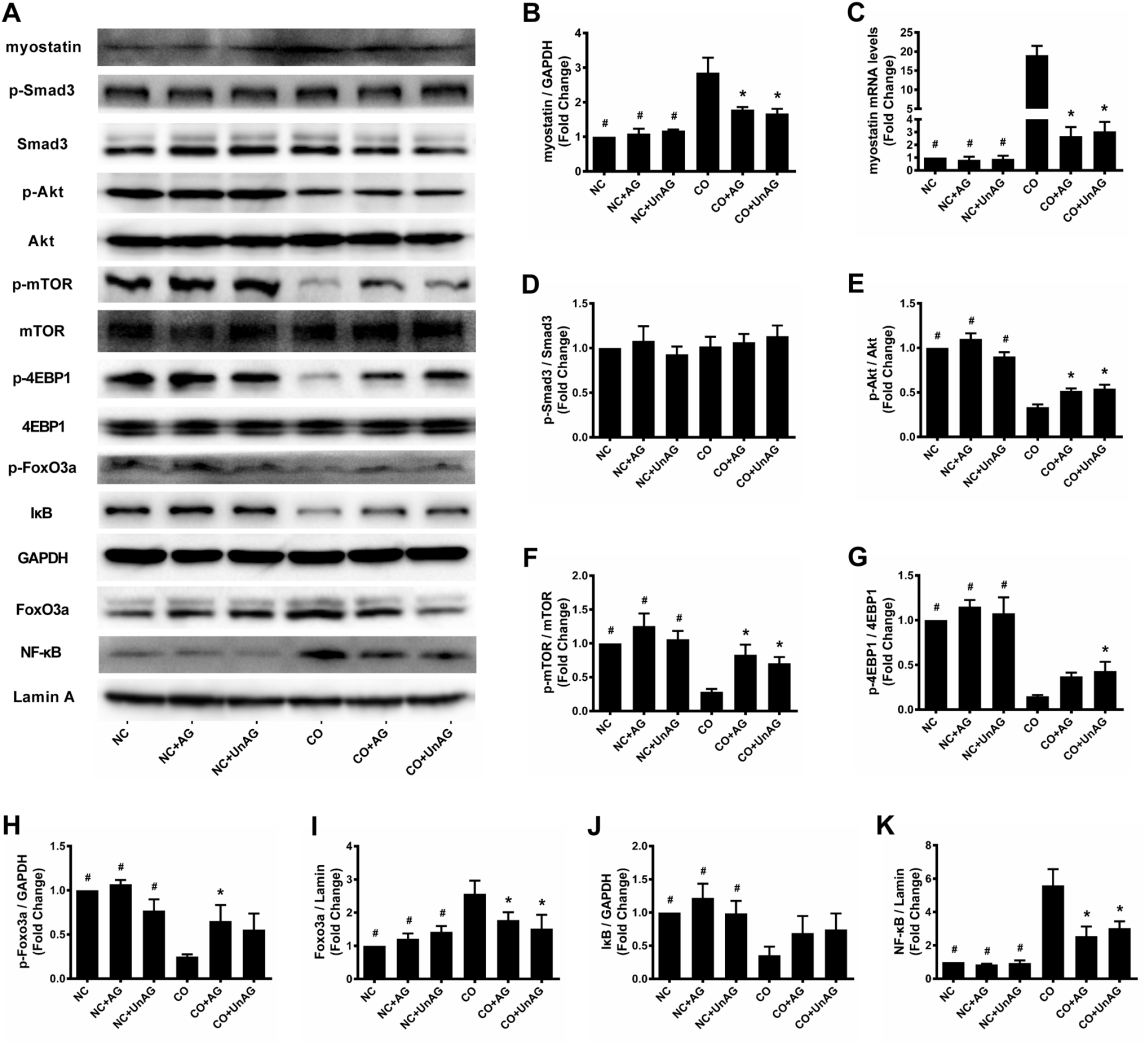
AG/UnAG decreased proteolytic markers and increased protein synthesis markers in co-cultured myotubes **(A)** Western blot of protein levels. **(B)** Quantification of myostatin was normalized to GAPDH. Significant differences were detected between CO and any NC groups (^#^P < 0.001), between CO and CO+AG/UnAG groups (*P < 0.001), by one-way ANOVA followed by Tukey test. **(C)** The levels of myostatin mRNA in C2C12 myotubes. mRNA levels were normalized to GAPDH. Significant differences were detected between CO and any NC groups (^#^P < 0.001), between CO and CO+AG/UnAG groups (*P < 0.001), by one-way ANOVA followed by Tukey test. **(D)** Quantification of p-Smad3 was normalized to total Smad3. No significant differences were detected between six groups, by one-way ANOVA followed by Tukey test. **(E)** Quantification of p-Akt was normalized to total Akt. Significant differences were detected between CO and any NC groups (^#^P < 0.001), between CO and CO+AG/UnAG groups (*P = 0.001, P < 0.001; respectively), by one-way ANOVA followed by Tukey test. **(F)** Quantification of p-mTOR was normalized to total mTOR. Significant differences were detected between CO and any NC groups (^#^P < 0.001), between CO and CO+AG/UnAG groups (*P = 0.001, P = 0.009; respectively), by one-way ANOVA followed by Tukey test. **(G)** Quantification of p-4EBP1 was normalized to total 4EBP1. Significant differences were detected between CO and any NC groups (^#^P < 0.001), between CO and CO+UnAG groups (*P = 0.025), by one-way ANOVA followed by Tukey test. **(H)** Quantification of p-FoxO3a was normalized to GAPDH. Significant differences were detected between CO and any NC groups (^#^P < 0.001, P < 0.001, P = 0.001), between CO and CO+AG groups (*P = 0.014), by one-way ANOVA followed by Tukey test. **(I)** Quantification of FoxO3a was normalized to Lamin A. Significant differences were detected between CO and any NC groups (^#^P < 0.001, P = 0.001, P = 0.002), between CO and CO+AG/UnAG groups (*P = 0.033, P = 0.005; respectively), by one-way ANOVA followed by Tukey test. **(J)** Quantification of IκB was normalized to GAPDH. Significant differences were detected between CO and any NC groups (^#^P = 0.015, P = 0.002, P = 0.017), by one-way ANOVA followed by Tukey test. **(K)** Quantification of NF-κB was normalized to Lamin A. Significant differences were detected between CO and any NC groups (^#^P < 0.001), between CO and CO+AG/UnAG groups (*P < 0.001), by one-way ANOVA followed by Tukey test. Data are represented as mean ± SD.

The ELISA results described above indicate that AG/UnAG prevented co-culture-induced increases in myostatin concentrations. Consistent with this result, AG/UnAG also prevented co-culture-induced increases in myostatin mRNA and protein levels (Figure 4A-4C). However, no significant differences in p-Smad3 and Smad3 protein levels were detected among the six groups (Figure 4A and 4D).

### PI3K/Akt and Akt/FoxO3a pathway activity

The PI3K/Akt signaling pathway regulates muscle mass by promoting protein synthesis and inhibiting protein degradation. To assess PI3K/Akt pathway activity, we measured Akt, p-Akt, mTOR, p-mTOR, 4EBP1, and p-4EBP1 protein levels in myotubes. As shown in Figure 4E-4G, co-culture decreased p-Akt, p-mTOR, and p-4EBP1 levels (*P* < 0.001), and AG/UnAG attenuated these decreases with the exception of p-4EBP1 levels in the CO+AG group (*P* = 0.092). Akt, mTOR, and 4EBP1 levels did not differ among the six different groups.

Akt phosphorylates and inhibits the nuclear translocation of FoxO3a, and decreased Akt activity promotes nuclear translocation of FoxO3a, which increases its transcriptional activity [26]. We therefore assessed FoxO3a activity using western blot assays and found that myotubes with lower p-Akt levels also had decreased cytoplasmic and increased nuclear FoxO3a levels (Figure 4A, 4H, and 4I).

### NF-κB activity

Accumulating evidence indicates that various stimuli contribute to muscle wasting by activating NF-κB [14]. To examine NF-κB activity in myotubes, nuclear extracts were analyzed using western blots. As shown in Figure 4A and 4K, co-culture increased nuclear NF-κB content, and AG/UnAG attenuated this increase. We also measured levels of cytoplasmic IκB, an NF-κB-binding protein whose degradation promotes nuclear translocation of NF-κB. As shown in Figure 4J, co-culture decreased cytoplasmic IκB levels; AG/UnAG tended to attenuate this decrease, but this effect was not statistically significant.

### Atrogin-1 and MuRF1 expression

**Figure 5:**
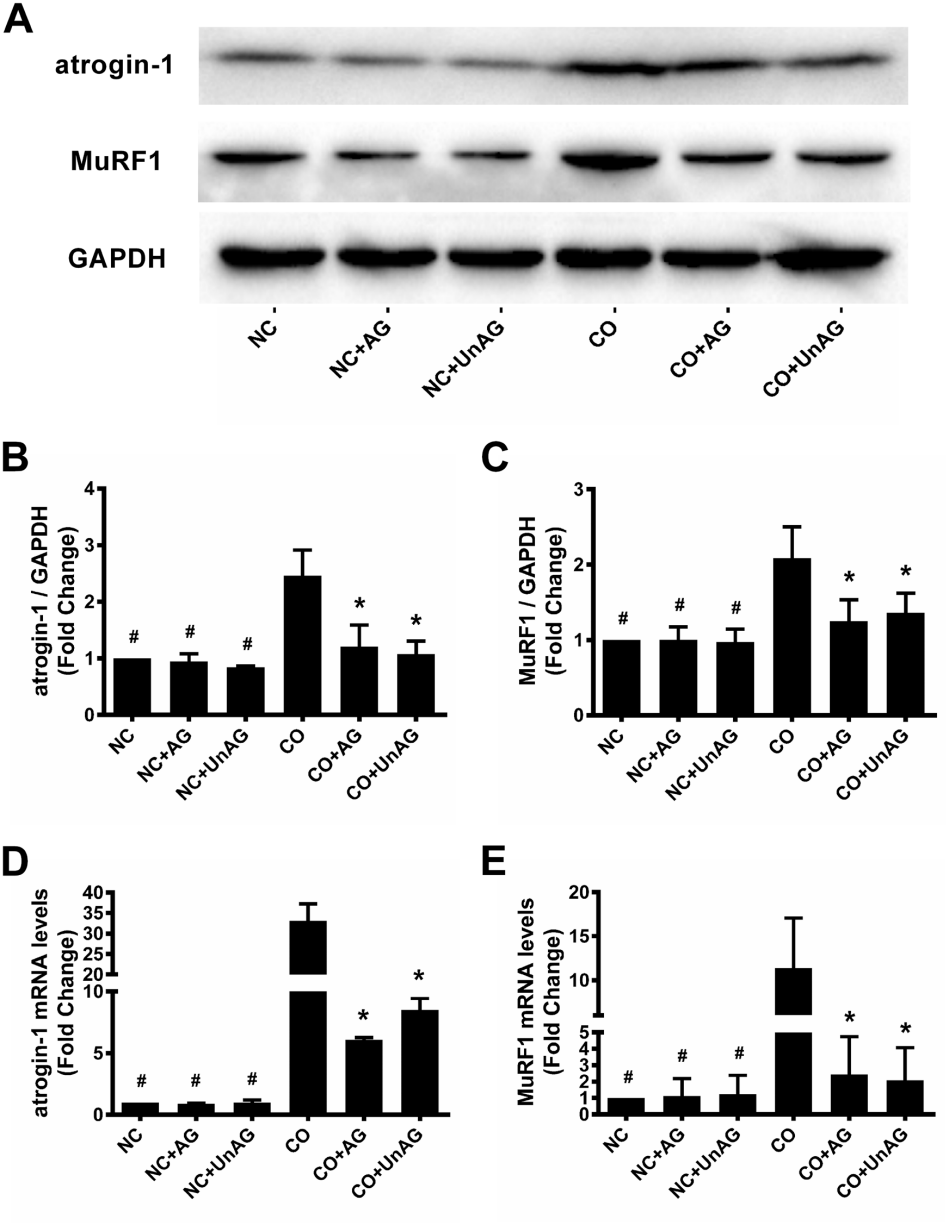
AG/UnAG attenuates ubiquitin E3 legases expression in co-cultured myotubes **(A)** Western blot of atrogin-1, MuRF1 and GAPDH in C2C12 myotubes. **(B)** Quantification of atrogin-1 was normalized to GAPDH. Significant differences were detected between CO and any NC groups (^#^P < 0.001), between CO and CO+AG/UnAG groups (*P = 0.001, P < 0.001; respectively), by one-way ANOVA followed by Tukey test. **(C)** Quantification of MuRF1 was normalized to GAPDH. Significant differences were detected between CO and any NC groups (^#^P = 0.002), between CO and CO+AG/UnAG groups (*P = 0.015, P = 0.037; respectively), by one-way ANOVA followed by Tukey test. **(D)** The levels of atrogin-1 mRNA in C2C12 myotubes. mRNA levels were normalized to GAPDH. Significant differences were detected between NC and CO groups (^#^P = 0.024), between CO and CO+AG/UnAG groups (*P = 0.034, P = 0.033; respectively), by one-way ANOVA followed by Dunnett's T3 test. **(E)** The levels of MuRF1 mRNA in C2C12 myotubes. mRNA levels were normalized to GAPDH. Significant differences were detected between NC and CO groups (^#^P = 0.006, P = 0.006; P = 0.007 respectively), between CO and CO+AG groups (*P = 0.016, P = 0.012), by one-way ANOVA followed by Tukey test. Data are represented as mean ± SD.

The muscle-specific ubiquitin ligases atrogin-1 and MuRF1 promote muscle protein degradation in several models of muscle atrophy [19]. We assessed the expression of these ligases using western blots and RT-qPCR. As shown in Figure 5A-5E, co-culture decreased atrogin-1 and MuRF1 mRNA and protein levels, and AG/UnAG prevented these decreases.

### Expression of μ-calpain, m-calpain, calpain-3, and calpastatin

**Figure 6:**
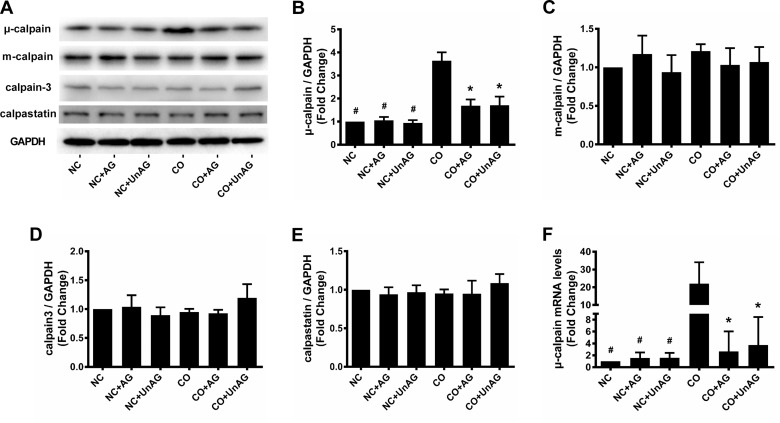
AG/UnAG attenuates calpain system activity in co-cultured myotubes **(A)** Western blot of μ-calpain, m-calpain, calpain-3, calpastatin and GAPDH in C2C12 myotubes. **(B)** Quantification of μ-calpain was normalized to GAPDH. Significant differences were detected between CO and any NC groups (^#^P < 0.001), between CO and CO+AG/UnAG groups (*P < 0.001), by one-way ANOVA followed by Tukey test. **(C-E)** Quantification of m-calpain, calpain-3 and calpastatin was normalized to GAPDH. No significant differences were detected among the six groups, by one-way ANOVA followed by Tukey test. **(F)** The levels of μ-calpain mRNA in C2C12 myotubes. mRNA levels were normalized to GAPDH. Significant differences were detected between NC and CO groups (^#^P = 0.005, P = 0.007; P = 0.007 respectively), between CO and CO+AG groups (*P = 0.01, P = 0.015 respectively), by one-way ANOVA followed by Tukey test. Data are represented as mean ± SD.

We recently demonstrated that the presence of tumors increased calpain activity and decreased calpastatin expression in the skeletal muscle of cachexic mice [23]. Here, we performed western blot and RT-qPCR studies to investigate whether co-culture and AG/UnAG affected the expression of calpains and calpastatin in myotubes. As shown in Figure 6A, 6B, and 6F, co-culture increased μ-calpain mRNA and protein levels (*P* < 0.01), and AG/UnAG inhibited these increases (*P* < 0.05). No significant differences in m-calpain, calpain-3, or calpastatin protein levels were observed among the six groups (Figure 6A and 6C-6E).

### Autophagy activity

**Figure 7:**
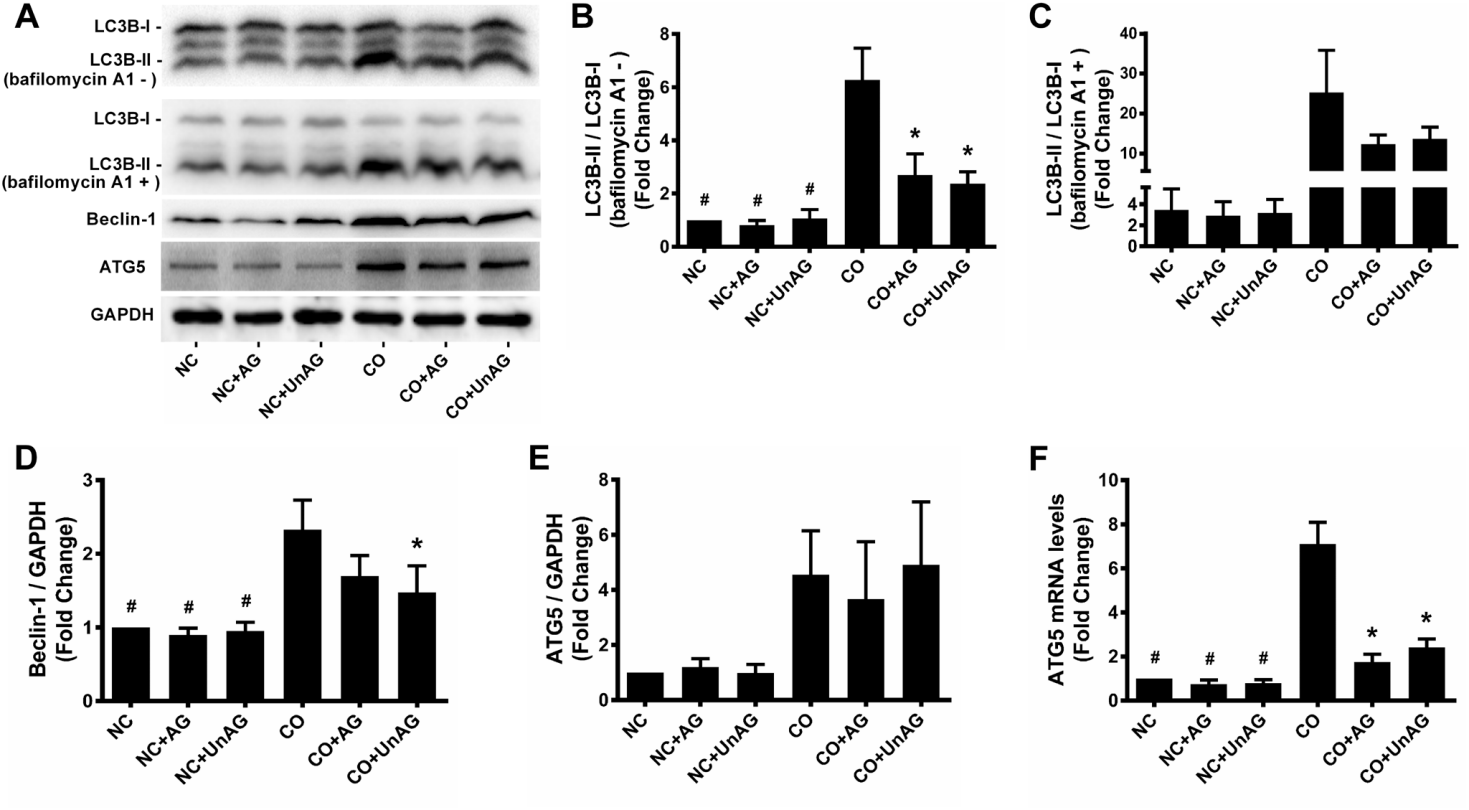
AG/UnAG attenuates autophagy activity in co-cultured myotubes **(A)** Western blot of LC3B-II/I, Beclin-1, ATG5 and GAPDH in C2C12 myotubes. **(B-C)** Quantification of LC3B-II/I ratio. In myotubes without bafilomycin A1 administration, significant differences were detected between CO and any NC groups (^#^P < 0.001), between CO and CO+AG/UnAG groups (*P < 0.001), by one-way ANOVA followed by Tukey test. Comparing with myotubes with and without bafilomycin A1 administration, no significant differences were detected among the twelves groups, by one-way ANOVA followed by Dunnett's T3 test. **(D)** Quantification of Beclin-1 was normalized to GAPDH. Significant differences were detected between CO and any NC groups (^#^P < 0.001), between CO and CO+UnAG groups (*P = 0.015), by one-way ANOVA followed by Tukey test. **(E)** Quantification of ATG5 was normalized to GAPDH. No significant differences were detected among the six groups, by one-way ANOVA followed by Dunnett's T3 test. **(F)** The levels of ATG5 mRNA in C2C12 myotubes. mRNA levels were normalized to GAPDH. Significant differences were detected between CO and any NC groups (^#^P < 0.001), between CO and CO+AG/UnAG groups (*P < 0.001), by one-way ANOVA followed by Tukey test. Data are represented as mean ± SD.

Activation of autophagy contributes to muscle wasting [7, 27]. We performed western blot and RT-qPCR studies to assess autophagy activity in myotubes. As shown in Figure 7A, 7B, and 7D, co-culture increased Beclin-1 protein levels and the LC3B-II/I ratio (*P* < 0.001); AG/UnAG inhibited these increases with the exception of Beclin-1 protein levels in the CO+AG group (*P* = 0.088). While co-culture tended to increase ATG5 protein levels, these increases did not reach statistical significance; in addition, no significant differences in ATG5 protein level were observed between the CO, CO+AG, and CO+UnAG groups (Figure 7E). However, co-culture increased ATG5 mRNA levels (*P* < 0.001), and AG/UnAG attenuated that increase (*P* < 0.001, Figure 7F).

Autophagy is a dynamic process, and accumulation of LC3B-II/I can result from either increased autophagic sequestration or decreased autophagosome clearance. We therefore performed a flux experiment in which myotubes were incubated with bafilomycin A1, a vacuolar H^+^-ATPase inhibitor that blocks lysosomal proteolysis, to inhibit autophagosome clearance [28]. As shown in Figure 4C, LC3B-II/I levels increased to a greater degree in myotubes incubated with bafilomycin A1, indicating that increased autophagic activity was responsible for the increased LC3B-II/I ratio; AG/UnAG attenuated this increased autophagic activity.

## DISCUSSION

Cancer cachexia is a life-threatening syndrome for which there is no effective therapy. Ghrelin is a multifunctional anabolic circulating hormone with anti-atrophic effects both *in vivo* and *in vitro* [29]. Here, AG/UnAG inhibited co-culture-induced decreases in myotube diameter and MHC2/MHC7 protein levels, indicating that AG/UnAG reduced co-culture-induced myotube atrophy.

We also found that AG/UnAG inhibited co-culture-induced downregulation of MHC mRNA expression, which suggests that AG/UnAG might inhibit myotube atrophy by promoting MHC protein synthesis. Indeed, AG/UnAG promoted the expression of the pro-myogenesis transcription factors myogenin and MyoD. Moreover, consistent with previous results [14], we found that AG/UnAG increased the activity of the critical anabolic PI3K/Akt pathway by promoting phosphorylation of Akt, mammalian target of rapamycin (mTOR) and eukaryotic translation initiation factor 4E binding protein 1 (4EBP1) in co-cultured myotubes. Akt also phosphorylates mTOR to increase its activity [30], which in turn increases 70 KD ribosomal S6 kinase (p70S6K) activity and promotes protein synthesis. In addition to activating p70S6 kinase, activated mTOR phosphorylates 4EBP1, a negative regulator of the protein initiation factor eIF-4E, to inhibit its activity [31]. Taken together, these findings indicate that AG/UnAG protects myotubes from co-culture-induced atrophy by promoting protein synthesis.

Atrogin-1 and MuRF1 are critical muscle-specific ubiquitin E3 ligases that mediate protein degradation. In addition, activation of NF-κB and FoxO3a up-regulates the expression of pro-inflammatory cytokines [14, 15, 21]. Consistent with these findings, we found that increased TNF-α concentration in the co-culture medium up-regulated FoxO3a activity, as indicated by increased nuclear Foxo3a levels and decreased cytoplasmic p-Foxo3a levels, and NF-κB activity, as indicated by increased nuclear P65 levels and decreased cytoplasmic IκB levels, in myotubes. Activated FoxO3a and NF-κB in turn promoted myotube atrophy by increasing atrogin-1 and MuRF1 expression.

Previous studies demonstrated that AG/UnAG inhibits the secretion of pro-inflammatory cytokines and subsequent activation of FoxO3a and NF-κB, thus downregulating atrogin-1 and MuRF1 expression and slowing muscle wasting [5, 13]. Consistent with these findings, we observed that AG/UnAG inhibited co-culture-induced increases in TNF-α concentrations and attenuated increased FoxO3a and NF-κB activity in co-cultured myotubes. AG/UnAG administration also inhibited increases in atrogin-1 and MuRF1 mRNA and protein levels in co-cultured myotubes. These data suggest that AG/UnAG reduces myotube atrophy by inhibiting the secretion of TNF-α. Previous studies demonstrated that AG/UnAG directly activates Akt, which in turn phosphorylates FoxO3a to inhibit its activity, and inhibits NF-κB activity in skeletal muscle [14, 15]. Our results indicate that AG/UnAG might also reduce atrophy by directly inhibiting FoxO3a and NF-κB activity, and thus down-regulating atrogin-1 and MuRF1 expression, in myotubes.

Calpain-dependent cleavage of myofilaments occurs upstream of the ubiquitin-proteasome pathway and is the initial step in myofilament degradation [19, 32]. μ-calpain and m-calpain are activated in atrophic muscles of patients with numerous conditions, such as cancer, sepsis, uremia, and burn injuries [33–36]. Here, we found that the muscle calpain system was activated in cachexic tumor-bearing mice, and calpain inhibitors could alleviate muscle wasting. Calpains activate FoxO3a, NF-κB, atrogin-1, and MuRF1 and inhibit Akt activity to induce muscle wasting [19–22]. Because ghrelin and calpains have similar downstream signal pathways, we investigated whether extrinsic ghrelin affected the myotube calpain system. Our results indicate that co-culture up-regulated μ-calpain expression in myotubes, and AG/UnAG inhibited this upregulation. In contrast, m-calpain, calpain-3, and calpastatin protein levels were not affected by co-culture or AG/UnAG. Taken together, these data demonstrate that co-culture activated the calpain system, as indicated by an increased calpain/calpastatin ratio, in myotubes, and AG/UnAG impaired this activation. AG/UnAG thus protects myotubes from atrophy by inhibiting calpain system activity.

Autophagy, which is inhibited by ghrelin, is a key contributor to muscle atrophy [16]. In this study, we observed that AG/UnAG attenuated increases in Beclin-1 protein levels, an early indicator of autophagy, and LC3B-II/I ratio, an indicator of autophagosome abundance, in co-cultured myotubes. These data suggest that AG/UnAG down-regulates co-culture-induced increases in autophagy activity in myotubes. AG/UnAG also attenuated increases in mRNA levels of ATG5, an essential mediator of autophagosome formation [28], in co-cultured myotubes. However, ATG5 protein levels did not differ between the CO, CO+AG, and CO+UnAG groups. This might be due to decreased μ-calpain activity in AG/UnAG-treated co-cultured myotubes, as μ-calpain can cleave ATG5 protein [37]. Taken together, these data indicate that AG/UnAG down-regulates autophagy activity to protect myotubes from atrophy.

Myostatin is a myogenesis inhibitor which is synthesized and secreted mainly by muscle tissue. Myostatin inhibits muscle protein synthesis by down-regulating myogenin and MyoD expression and inhibiting Akt activity via the ActRIIb/Smad3 pathway [38]. Moreover, it promotes muscle protein degradation via the ActRIIb/Smad3 pathway by up-regulating atrogin-1 expression [25]. Similar to a previous study in which ghrelin inhibited an increase in myostatin expression in skeletal muscle [39], our study revealed that AG/UnAG inhibited increases in myostatin levels in co-culture medium and co-cultured myotubes. These results indicate that AG/UnAG attenuated increases in myostatin synthesis and secretion in co-cultured myotubes. However, neither Smad3 nor p-Smad3 levels differed among the six groups, indicating that Smad3 signaling activity was unaffected. The observed changes in levels of downstream Smad3 substrates might therefore be mediated by other signal pathways or another myostatin-dependent mechanism. Additional studies are needed to identify this mechanism.

Cancer cachexia causes nearly 20% of all cancer-related deaths [2], and its pathogenesis is not completely understood. Although some tumor-bearing animal models have been developed to study cancer cachexia [5, 7], to the best of our knowledge, cell co-culture models have not been used to simulate cancer cachexic muscle atrophy. Our cell co-culture model uses a Transwell system to grow two types of cells in the same culture medium allows intercellular communications via cellular secretions. This model has been used to investigate cell-cell interactions between multiple cell types, such as adipocytes and skeletal muscle fibers [40] and osteoblast and mesenchymal stromal cells [41]. In this study, co-culture of C2C12 myotubes with CT26 colon carcinoma cells increased TNF-α and myostatin concentrations in the medium and altered the metabolism of C2C12 myotubes, indicating that these two types of cells interact with each other via secreted factors. Muscle atrophy associated with cancer cachexia results from tumor-host interactions mediated by pro-inflammatory cytokines and pro-cachectic factors. Moreover, increased TNF-α and myostatin concentrations are often detected in tumor-bearing animals and cancer cachexic patents [2, 7, 24]. Such findings suggest that our co-culture model can, at least in part, simulate the cancer cachexic environment *in vitro*. Because many other circulating factors are involved in cancer cachexia, additional studies are needed to investigate whether these factors are involved in our co-culture model.

To the best of our knowledge, we demonstrated here for the first time that AG/UnAG inhibits calpain system activity. However, the exact signaling mechanisms underlying this inhibition remain unknown. Previous studies have shown that both AG and UnAG can act directly on skeletal muscle, which contains many high-affinity binding sites [14, 15, 42]. However, the identity of the AG/UnAG receptor also remains unknown and requires further investigation. Because increases in apoptosis contribute to muscle wasting [32] and AG/UnAG has anti-apoptotic effects [43], future studies should examine whether co-culture of myotubes with CT26 cells induces myotube apoptosis, and if so, how AG/UnAG affects that apoptotic process.

In conclusion, we demonstrated that co-culture of C2C12 myotubes with CT26 colon carcinoma cells increased TNF-α and myostatin concentrations in the culture medium. Additionally, co-culture increased protein degradation and decreased protein synthesis in the myotubes via multiple mechanisms. AG/UnAG inhibited these effects and protected co-cultured myotubes against atrophy. While the specific anti-atrophic mechanisms of AG/UnAG have not been fully elucidated, we utilized a novel *in vitro* model to investigate cancer cachexic muscle atrophy, and our results may contribute to the development of an AG/UnAG-based treatment for that disorder.

## MATERIALS AND METHODS

### Cell culture

Mouse C2C12 myoblasts and CT26 colon carcinoma cells were obtained from ATCC (Manassas, VA, USA) and maintained in growth medium composed of Dulbecco's Modified Eagle's Medium (DMEM) (Invitrogen, Carlsbad, CA, USA) supplemented with 10% fetal bovine serum (FBS) (Gibco, New Zealand) and 1% penicillin-streptomycin (Invitrogen) in an atmosphere of 5% CO_2_ at 37°C.

To induce differentiation, C2C12 myoblasts were seeded in the lower wells of a 6-well Transwell-Clear plate (Corning, #3450) at a density of 30,000 cells/cm^2^ and cultured in growth medium for 24-48 hours. When the myoblasts reached 90-100% confluence, the medium was replaced by a differentiation medium composed of DMEM supplemented with 2% horse serum (Gibco, New Zealand) and 1% penicillin-streptomycin, and the myoblasts were cultured for another 4 days to allow their differentiation into myotubes. The differentiation medium was changed daily. The differentiation medium was then replaced by growth medium and CT26 cells were seeded (20,000 cells/cm^2^) into the upper inserts (0.4 μm pore polyester membrane) of another 6-well plate (Corning, #3516) that contained growth medium. After 24 h of culture, the upper inserts were placed into the lower wells containing myotubes. The base of each insert contained a membrane with 0.4 μm pores that allowed the movement of secreted factors, and thus permitted paracrine interactions to occur between the two different cell types. Next, both the lower well and upper insert medium was changed to growth medium with or without AG/UnAG (TOCRIS, 1465/2951, 100 nM). After 24 h of co-culture, the myotubes were harvested for RT-qPCR and western blot analysis or fixed for immunocytochemistry studies. For autophagic flux measurements, bafilomycin A1 (Santa Cruz Biotechnology; Santa Cruz, CA USA; sc-201550A, 200 nM) was added to the medium 4 h before harvesting the myotubes, as previously described [7].

The co-culture combinations consisted of sham myotubes (without CT26 cells in the insert) without ghrelin (NC group); sham myotubes with acylated ghrelin (NC+AG group); sham myotubes with unacylated ghrelin (NC+UnAG group); myotubes with CT26 cells but without ghrelin (CO group); myotubes with CT26 cells and acylated ghrelin (CO+AG group); myotubes with CT26 cells and unacylated ghrelin (CO+UnAG group).

### Immunocytochemical analysis

Myotubes were fixed in PBS with 4% paraformaldehyde for 15 min at room temperature and then permeabilized in PBS containing 0.2% Triton X-100 for 5 min at room temperature. The myotubes were then blocked with PBS containing 5% goat serum and 2% BSA for 1 h at room temperature and then incubated overnight at 4°C with anti-MHC antibody (Abcam; Cambridge, UK; ab91506, 1:200). Next, the myotubes were incubated with Alexa Fluor 488-conjugated goat anti-rabbit IgG (Abcam; ab150077, 1:500) for 1 h at room temperature and then mounted with Fluoroshield Mounting Medium containing DAPI (Abcam; ab104139). Images were acquired with a fluorescence microscope (Nikon; Tokyo, Japan) and analyzed with Image J software (version 1.46r, NIH, USA).

Myotube diameters were measured as previously described [38]. Briefly, 20 pictures were taken per well, and the diameters of the five largest myotubes (those containing ≥ 3 nuclei when viewed at ×100 magnification) in each picture were measured. Next, the mean diameter of a single myotube was calculated based on three independent measurements. The measurement points were separated by 200 μm. This method was also used to calculate the mean diameter ± SD of the 100 largest myotubes in each well.

### Western blot analysis

Myotubes were washed two times in ice-cold PBS and then transferred to 4°C cell lysis reagent (20 mM Tris-HCl (pH 7.5), 150 mM NaCl, 1 mM Na_2_EDTA, 1 mM EGTA, 1% Triton, 2.5 mM sodium pyrophosphate, 1 mM beta-glycerophosphate, 1 mM Na_3_VO_4_, 1 μg/mL leupeptin) supplemented with a protease inhibitor cocktail (Roche, 05892970001). Protein concentrations were measured using a BCA kit (BCA1, Sigma-Aldrich, St Louis, MO, USA). Nuclear proteins were isolated using a CelLytic NuCLEAR Extraction Kit (NXTRACT, Sigma-Aldrich). Aliquots of total protein (20 μg/lane) were separated by electrophoresis on a 4-20% SDS-PAGE gel, and the separated protein bands were transferred onto PVDF membranes. The membranes were blocked with Tris-buffered saline-0.1% Tween-20 (TBST) containing 5% skim milk, incubated overnight at 4°C with a primary antibody in TBST containing 5% BSA, and then incubated with a HRP-conjugated anti-rabbit secondary antibody (Abcam, ab97051, 1:2000). The immunostained proteins were visualized with enhanced chemiluminescence reagents (GE2301, Gen-View Scientific, Arcade, NY, USA). Images of the membranes were recorded with a ChemiDoc XRS+ system (Bio-Rad, Hercules, CA, USA), and analyzed using Quantity One software (version 4.6.6, Bio-Rad, USA). The following antibodies were used as primary antibodies: Abcam: anti-MHC2 (ab124937); anti-MHC7 (ab172967); anti-myogenin (ab124800); anti-MyoD (ab64159); anti-myostatin (ab98337); anti-IκB (ab32518); anti-NFκB P65 (ab32536); anti-atrogin-1 (ab168372); anti-MuRF1 (ab172479); anti-u-calpain (ab108400); anti-m-calpain (ab126600); anti-calpastatin (ab28252); anti-Beclin1 (ab207612); anti-LC3B (ab48394); anti-ATG5 (ab109490); anti-Akt (phospho S473, ab81283); anti-Akt (ab179463); anti-mTOR (phospho S2448, ab109268); anti-mTOR (ab32028); anti-4EBP1 (phospho T37, ab75767); anti-4EBP1 (ab32024); anti-Smad3 (phospho S423 + S425, ab52903); anti-Smad3 (ab40854); anti-FoxO3a (phospho S253, ab31109); anti-FoxO3a (ab70315); anti-Lamin A (ab26300); anti-GAPDH (ab181602); Santa Cruz Biotechnology: anti-calpain-3 (sc-365277).

### Real-time quantitative RT-PCR analysis

Total RNA was extracted from myotubes with Trizol reagent (Invitrogen, 15596026) according to the manufacturer's instructions. Reverse transcription was performed with Super Script II reverse transcriptase (Invitrogen, 18064022). The resulting cDNA for specific transcripts was used for real-time quantitative PCR (RT-qPCR) performed with PowerUp SYBR Green Master Mix (A25742, Life Technologies; Carlsbad, CA, USA) and a 7500 Real-time PCR system (Applied Biosystems; Foster City, CA, USA). Gene expression data was normalized to that of a housekeeper gene (GAPDH). Relative gene expression levels were obtained using the 2^−ΔΔCT^ method. The RT-qPCR primer sequences used are listed in Table 1.

**Table 1:** Primers used for qPCR

Gene	Forward	Reverse
MHC	5′-CGGCAATGAGTACGTCACCAAA −3′	5′-TCAAAGCCAGCGATGTCCAA-3′
myogenin	5′-CGTGGGCATGTAAGGTGTGTAAGA −3′	5′-CATTCACTTTCTTGAGCCTGCGCT-3′
MyoD	5′-GAGGATCCGATGGAGCTTCTATCG −3′	5′-CGGATCCTCTCAAAGCACCTGATA-3′
myostatin	5′-GGGCATGATCTTGCTGTAACCTTC −3′	5′-CGTGGAGTGTTCATCACAGTCAAG-3′
atrogin-1	5′-GTCGCAGCCAAGAAGAGAAAGA −3′	5′-TGCTATCAGCTCCAACAGCCTT-3′
MuRF1	5′-TAACTGCATCTCCATGCTGGTG −3′	5′-TGGCGTAGAGGGTGTCAAACTT-3′
μ-calpain	5′-CATGGCTAAGAGCAGGAAGG −3′	5′-CGAAGTCTGCAGGTCTAGGG-3′
Atg5	5′-ATCAGACCACGACGGAGCGG −3′	5′-GGCGACTGCGGAAGGACAGA-3′
GAPDH	5′-ATGACAATGAATACGGCTACAGCAA-3′	5′-GCAGCGAACTTTATTGATGGTATT-3′

MHC, myosin heavy chain; MuRF1, muscle ring finger 1; Atg5, autophagy related gene 5; GAPDH, glyceraldehyde-3-phosphate dehydrogenase.

### ELISA

Mouse TNF alpha (Abcam, ab46105), mouse IL-1 beta (Abcam, ab100704), and GDF-8/Myostatin Quantikine (R&D Systems, Minneapolis, MN, USA, DGDF80) ELISA kits were used to measure TNF-α, IL-1β, and myostatin concentrations, respectively, in cell culture medium according to the manufacturer's instructions. The assay plates were read using a SpectraMax M5 microplate reader (Molecular Devices; Sunnyvale, CA, USA).

### Statistical analysis

Each experiment was repeated at least three times, and all data were analyzed using IBM SPSS Statistics for Windows, Version 19.0 (Armonk, NY; IBM Corp). Results are shown as means ± SD. Statistical comparisons between groups were performed using one-way ANOVAs followed by the Tukey test when equal variances were assumed. When equal variances were not assumed, Dunnett's T3 test was applied. Two-sided *P*-values < 0.05 were considered statistically significant.
